# Epidemiology of Antimicrobial Resistance in *Escherichia coli* Isolates from Raccoons (*Procyon lotor*) and the Environment on Swine Farms and Conservation Areas in Southern Ontario

**DOI:** 10.1371/journal.pone.0165303

**Published:** 2016-11-09

**Authors:** Kristin J. Bondo, David L. Pearl, Nicol Janecko, Patrick Boerlin, Richard J. Reid-Smith, Jane Parmley, Claire M. Jardine

**Affiliations:** 1 Department of Pathobiology, University of Guelph, Guelph, Ontario, Canada; 2 Department of Population Medicine, University of Guelph, Guelph, Ontario, Canada; 3 Centre for Foodborne, Environmental and Zoonotic Infectious Diseases, Public Health Agency of Canada, Guelph, Ontario, Canada; 4 Department of Biology and Wildlife Diseases, University of Veterinary and Pharmaceutical Sciences Brno, Brno, Czech Republic; 5 Canadian Wildlife Health Cooperative, Department of Pathobiology, University of Guelph, Guelph, Ontario, Canada; USDA-ARS Salinity Laboratory, UNITED STATES

## Abstract

Antimicrobial resistance is a global threat to livestock, human and environmental health. Although resistant bacteria have been detected in wildlife, their role in the epidemiology of antimicrobial resistance is not clear. Our objective was to investigate demographic, temporal and climatic factors associated with carriage of antimicrobial resistant *Escherichia coli* in raccoons and the environment. We collected samples from raccoon paws and feces and from soil, manure pit and dumpsters on five swine farms and five conservation areas in Ontario, Canada once every five weeks from May to November, 2011**–**2013 and tested them for *E*. *coli* and susceptibility to 15 antimicrobials. Of samples testing positive for *E*. *coli*, resistance to ≥ 1 antimicrobials was detected in 7.4% (77/1044; 95% CI, 5.9–9.1) of raccoon fecal samples, 6.3% (23/365; 95% CI, 4.0–9.3) of paw samples, 9.6% (121/1260; 8.0–11.4) of soil samples, 57.4% (31/54; 95% CI, 43.2–70.8) of manure pit samples, and 13.8% (4/29; 95% CI, 3.9–31.7) of dumpster samples. Using univariable logistic regression, there was no significant difference in the occurrence of resistant *E*. *coli* in raccoon feces on conservation areas versus farms; however, *E*. *coli* isolates resistant to ≥ 1 antimicrobials were significantly less likely to be detected from raccoon paw samples on swine farms than conservation areas and significantly more likely to be detected in soil samples from swine farms than conservation areas. Resistant phenotypes and genotypes that were absent from the swine farm environment were detected in raccoons from conservation areas, suggesting that conservation areas and swine farms may have different exposures to resistant bacteria. However, the similar resistance patterns and genes in *E*. *coli* from raccoon fecal and environmental samples from the same location types suggest that resistant bacteria may be exchanged between raccoons and their environment.

## Introduction

Antimicrobial resistance (AMR) is a global public, livestock and environmental health concern [1, 2]. Selection pressure that occurs as a consequence of antimicrobial use, mainly in humans and animals, is a primary driver leading to the emergence of resistant bacteria [3, 4]. Although wildlife are generally not deliberately exposed to antimicrobials, resistant bacteria have been detected in the fecal bacteria of a variety of wild animals in various locations around the world [5–10].

To understand the potential sources of antimicrobial resistant bacteria, several studies have examined the prevalence of resistant bacteria in wildlife populations with varying levels of exposure to human activity and/or exposure to antimicrobial sources. Generally, but not universally, wild animals living in close proximity to humans and agriculture carry more resistant bacteria than those living in areas with little anthropogenic influence [10–18]. Conversely, wildlife living in the most remote areas of the world typically carry little to no antimicrobial resistant bacteria [14, 19–22].

Some anthropogenic environmental sources of resistant bacteria for wildlife include livestock manure, agricultural run-off, effluent from hospitals and sewage treatment plants, raw meat or other animal products [23], soil containing antimicrobial residues and resistant bacteria, pet feces, and antimicrobial use in aquaculture [24–27]. Contaminated fresh water also is a potential source of antimicrobials for wildlife and may play an important role in the transmission of AMR genes among bacteria and dissemination of resistant bacteria from livestock or humans [28].

Relatively little is known about the epidemiology of antimicrobial resistant bacteria in wildlife and in environments with different levels of human activity. Until recently, few studies have taken into consideration point-sources of antimicrobial or antimicrobial resistant bacterial pollution and sampled the environment in addition to wildlife. Consequently, some wildlife studies documenting high prevalence of AMR from sites that claimed to be pristine have been criticized because they have actually had a history of antimicrobial use in the past or are close to anthropogenic areas [21, 29, 30]. Many AMR studies have focused on mice and voles with small home ranges [9, 10, 13], but migratory birds [31] or wild mammals with larger home-ranges, such as raccoons, may be more involved in the widespread dissemination of antimicrobial resistant bacteria [32], and be better indicators of resistant bacteria present in the environment at the landscape level. Raccoons may also have the potential to mechanically transmit resistant bacteria on their paws [33]. Similarly, antimicrobial resistant bacteria also have been detected on the foot pads [34] and from external wash samples [35] of wild birds.

Using a repeated cross-sectional study conducted over 3 years, our objectives were to: (1) compare the prevalence and patterns of AMR and associated resistance genes in *Escherichia coli (E*. *coli*) isolates from raccoon and environmental samples from swine farms and conservation areas, and (2) assess the impact of season, climate, year, location type, and raccoon demographic factors on the occurrence of antimicrobial resistant *E*. *coli* in raccoon fecal and environmental samples. We predicted that raccoons and environmental samples from swine farm environments would have a higher prevalence of resistant bacteria than samples from conservation areas because of the potential exposure to antimicrobials added to livestock feed or water and to antimicrobial metabolites present in livestock manure that is commonly used as fertilizer and spread onto agricultural fields.

## Materials and Methods

Procedures for trapping and handling raccoons were approved by the Animal Care Committee at the University of Guelph following the guidelines of the Canadian Committee on Animal Care (Permit number: 11R015). The Ontario Ministry of Natural Resources and Forestry issued a permit to live-trap and collect samples from raccoons in this study, and permission to work on sites was granted by the Grand River Conservation Authority, the University of Guelph, and from private land owners as appropriate. The sites, trapping and sample collection methods used in this study have been described previously [36]. Briefly, raccoons were live-trapped on five swine farms and five conservation areas from May through November, 2011–2013. During this period, each site was trapped once every five weeks. All sites were located within the boundaries of the Grand River watershed in Ontario within a 100-km radius of either Guelph (43°32’42.32” N 80°15’01.87” W) or Cambridge (43°21’49.63” N 80°18’50.68” W). The distance between sites ranged from 1.3 to 52.2 km. The Grand River watershed is the largest watershed in Ontario and is approximately 6800 km^2^ [37].

All of the farm sites selected for this study identified themselves as being primarily swine farms and were part of FoodNet Canada, a sentinel site-based enteric pathogen surveillance program [38]. The farms were chosen based on their proximity to the University of Guelph and included farrow-to-young grower and farrow-to-finish operations. The attributes of the study sites have been described previously [36]. Three of the five farms did not administer injectable antimicrobials or antimicrobials in-feed to swine. The other two farms administered both injectable and in-feed antimicrobials to swine. The conservation areas were all located primarily in peri-urban areas and ranged in size from 75**–**1608 ha. Recreational activities included hiking, fishing, picnicking, camping and swimming in many of these areas.

### Sample Collection

Raccoons were live-trapped and processed as described previously [33]. Briefly, at each site 20**–**40 Tomahawk live traps (Tomahawk Live Trap Co. Tomahawk, Wisconsin, USA) were set 3–4 nights/trapping week at each site in areas with limited public access, but where raccoons were known to be present, including around dumpsters and buildings. Upon capture, raccoons were anesthetized using an intramuscular injection of 0.025 mg/kg dexmedetomidine hydrochloride (Dexdomitor 0.5 mg/ml; Pfizer Animal Health, Kirkland, Quebec, Canada) and 5 mg/kg ketamine hydrochloride (Vetalar 100 mg/ml; Bioniche Animal Health, Belleville, ON, Canada). A numbered metal ear tag (1005–3, National Band and Tag Co. Newport, Kentucky, USA) was then placed in one ear and a passive integrated transponder tag (GPT12 Pre-Load Sterile, Biomark, Boise, Idaho, USA) was injected subcutaneously between the shoulder blades for subsequent identification. Sex, age class (adult or juvenile, on the basis of animal size and teeth wear/staining), and body mass were recorded for each animal. Fecal swabs were collected per rectum using Cary-Blair applicators (BBL CultureSwab, BD; Becton, Dickinson and Company, Annapolis, Maryland, USA) and in 2012, paw samples were also collected using a Swiffer^®^ (Armstrong, Proctor and Gamble, Cincinnati, Ohio, USA) soaked in 25 ml of sterile saline as previously described [33]. Although individuals were only sampled once per trapping week, multiple samples were collected from the same individual if they were caught in subsequent trapping sessions.

Environmental sample collection has been previously described [36]. Briefly, ten to twenty soil samples were collected within 2 m of where traps were placed on the first day of each trapping week at each study site. Approximately 10 g of soil, free of obvious fecal contamination, was collected into sterile vials. At each swine farm, one manure pit sample was collected on the first day of each trapping week. During each trapping session of 2013, dumpster samples were collected when available from three conservation areas. Dumpster samples were collected < 1 day after dumpsters were emptied.

### Laboratory Work

All samples were submitted for *E*. *coli* isolation and antimicrobial susceptibility testing. The McEwen Group Research Lab at the Canadian Research Institute for Food Safety, University of Guelph, (Guelph, Ontario, Canada) cultured *E*. *coli* from samples, and the Canadian Integrated Program for Antimicrobial Resistance Surveillance (CIPARS) AMR Lab at the National Microbiology Laboratory of Guelph (formerly the Laboratory for Foodborne Zoonoses (LFZ)), Public Health Agency of Canada (Guelph, Ontario, Canada) conducted the susceptibility testing. Methods used have been previously described [32]. Up to three isolates of *E*. *coli* per sample were submitted for antimicrobial susceptibility in 2011, and only one isolate of *E*. *coli* per sample was submitted from 2012–2013.

The National Antimicrobial Monitoring System (NARMS) CMV2AGNF plate for susceptibility testing was used; it included 15 antimicrobials from the following seven antimicrobial classes: β-lactams [ampicillin (AMP), amoxicillin-clavulanic acid (AMC), cefoxitin (FOX), ceftiofur (TIO), and ceftriaxone (CRO)], aminoglycosides [streptomycin (STR), kanamycin (KAN), and gentamicin (GEN)], tetracyclines [tetracycline (TCY)], phenicols [chloramphenicol (CHL)], inhibitors of the folic acid pathway [sulfisoxazole (SOX) and trimethoprim-sulfamethoxazole (SXT)], macrolides [azithromycin (AZM)], and quinolones [nalidixic acid (NAL) and ciprofloxacin (CIP)]. The broth microdilution automated Sensititre^™^ System (TREK Diagnostics, Ohio, USA) was used for susceptibility testing, and Minimum Inhibitory Concentration (MIC) breakpoints were those used by CIPARS and NARMS, which are derived from Clinical Laboratory Standards Institute breakpoints [39]. Based on these breakpoints, isolates were classified as susceptible, intermediate or resistant. For the analysis, we considered all isolates classified as intermediate or resistant to be resistant and grouped the antimicrobials into the 7 classes described above.

Regardless of resistance pattern, all resistant and intermediate isolates were tested by the Boerlin Lab at the University of Guelph for the presence of major AMR genes using single and multiplex PCR as described previously [13, 40]. The resistance genes were as follows: *sul1*, *sul2*, *sul3* for inhibitors of the folic acid pathway*; tet*(A), *tet*(B), and *tet*(C) for tetracycline; *aadA*, *aadB*, *aphA1*, *aphA2*, *aac(3)IV*, and *strAB* for aminoglycosides; *bla*_TEM_, *bla*_SHV_, *bla*_OXA1_,*bla*_CTX-M_, and *bla*_CMY-2_ for β-lactams, and *catA1*, *cmlA*, and *floR* for phenicols. The *bla*_CTX-M_ genes were further sequenced to identify the subtype of CTX-Ms using methods described previously [41].

### Statistical Modeling

Mean daily temperature and total rainfall per day were downloaded from Environment Canada from the nearest weather station with complete data (Fergus Ministry of the Environment (MOE), ON) from 2011 to 2013. Missing values were filled in using data from the next nearest weather station in Guelph, Ontario. We investigated rainfall and temperature variables during three time periods prior to sample collection over the duration of each sampling period (3 days, 14 days, and 30 days) because survival and persistence of *E*. *coli* in soils from temperate climates is variable and has been reported to vary from a few days to months [42].

Linearity between the log odds of testing positive for *E*. *coli* resistant to ≥ 1 antimicrobial and the continuous independent variables were determined individually by examining a lowess curve and the significance of a quadratic term and its main effect in each multi-level model. Independent variables that had nonlinear relationships with the outcome variable based on a significant quadratic term and visual assessment of the lowess curve were categorized or modelled as a quadratic relationship if appropriate. Wald’s χ^2^ test was used to test the significance of categorical variables. For each model, the odds ratio and 95% confidence interval (CI) of each variable were reported.

If applicable, random effects were used to account for autocorrelation among antimicrobial resistant *E*. *coli* isolates taken from the same site, animal/manure pit/dumpster, and/or sample. Random effects were excluded from models if their inclusion explained very little of the variation, and if excluding them resulted in a model with a lower Akaike’s Information Criterion (AIC) and Bayesian Information Criterion (BIC) [43]. To determine the amount of variation explained by the site, animal, sample, and isolate level, variance partition coefficients (VPCs) were estimated from the variance components of each final model that included both fixed and random effects using the latent variable technique [43]. Pearson and deviance residuals were used to determine if there were any outlying observations, and best linear unbiased predictions (BLUPs) of the random effects were examined to assess model fit.

#### Univariable models

All statistical tests were conducted using STATA (STATA Intercooled 13.1; StataCorp, College Station, Texas, USA). For statistical modeling, univariable multi-level logistic regression was used to model: AMR to ≥ 1 antimicrobials in *E*. *coli* isolates from raccoon fecal, soil, and manure pit samples modeled separately; AMR to ≥ 1 antimicrobials in *E*. *coli* isolates from raccoon fecal, soil, manure pit, and dumpster samples with sample type as an independent variable; and AMR to ≥ 1 antimicrobials in *E*. *coli* isolates from raccoons of the same individual with source (paw or fecal samples) as an independent variable. Each sample type was analyzed separately except the dumpster samples due to small effective sample size.

Sample type was included as the only explanatory variable in the models comparing prevalence of antimicrobial resistant *E*. *coli* isolates between raccoon fecal and paw samples and among manure pit, dumpster, soil and fecal samples. For the models where sample types were analyzed separately, explanatory variables, if applicable, were raccoon sex (male or female), raccoon age (adult or juvenile), location type (swine farm or conservation area), year (2011–2013), sum of rainfall over 3, 14, or 30 days prior to sample collection, mean temperature over 3, 14, or 30 days prior to sample collection, and season. Two distinct seasons ecologically important for raccoons were considered: rearing (May–July) and pre-denning/dispersal (August–October) as defined by Rosatte et al. [44].

Univariable models examining the impacts of age, sex, season, and year on the occurrence of resistance to each of the five most common antimicrobials detected were created for raccoon fecal and soil samples. For raccoon fecal samples, the univariable models would not converge when a random effect was added to account for multiple isolates per sample, so the resistance results were pooled into one observation/sample for these analyses. There were 3/10 occasions in raccoon fecal samples where multiple resistant isolates were collected per sample, and the AMR phenotype results for the isolates did not match. In these cases, the resistance phenotype results from multiple isolates per sample were combined into one result. For example, if any one of the isolates was resistant to a particular antimicrobial, then the sample was considered to have resistance to that antimicrobial.

#### Multivariable models

After the univariable random effects models were constructed for raccoon fecal, raccoon paw, soil, and manure pit samples, multi-variable main effects models were explored. Due to small effective sample sizes, no interactions were tested for any of the sample types. To avoid issues with collinearity, separate multivariable models were created for each rainfall and temperature variable corresponding to each time period (e.g., 3, 14, 30 days). If the rainfall and temperature variables for each time period were correlated, | p | > 0.8, then the two variables were modelled separately to avoid issues with collinearity.

When creating multivariable models, a model including all potential main effects was initially constructed and variables that were not statistically significant were removed if they were not confounding variables; full and reduced models were compared using likelihood ratio tests. A variable was considered to be a confounding variable if it was a nonintervening variable and its removal from the model resulted in ≥ 30% change in the coefficients of a statistically significant variable [43]. Associations were considered significant at α = 0.05.

## Results

We collected 1606 soil, 31dumpster, and 69 manure pit samples from the environment, and 1095 fecal and 417 paw samples from 627 and 306 individual raccoons, respectively. Three individual raccoons moved between two sites, so we randomly selected and removed one fecal and the corresponding paw sample from each of those individuals from the analysis. Sex and age were not recorded for one and three raccoons, respectively.

### Detection of *E*. *coli*

*Escherichia coli* was isolated from 95.6% (1044/1092; 95% CI, 94.2–96.7) of raccoon fecal samples, 87.7% (365/416 95; 95% CI, 84.2–90.7) of paw samples, 78.4% (1260/1606; 95% CI, 76.4–80.4) of soil samples, 93.5% (29/31; 95% CI, 3.9–31.7) of dumpster samples, and 78.2% (54/69; 95% CI, 66.7–87.3) of manure pit samples.

### *E*. *coli* Susceptibility

Of samples testing positive for *E*. *coli*, resistance to ≥ 1 antimicrobials was detected in 7.4% (77/1044; 95% CI, 5.9–9.1) of raccoon fecal samples from 616 individuals, 6.3% (23/365; 95% CI, 4.0–9.3) of paw samples from 259 individuals, 9.6% (121/1260; 8.0–11.4) of soil samples, 57.4% (31/54; 95% CI, 43.2–70.8) of manure pit samples, and 13.8% (4/29; 95% CI, 3.9–31.7) of dumpster samples. The proportion of *E*. *coli* positive samples resistant to ≥1 antimicrobials or antimicrobial classes in each location type are presented by sample type in Table 1. The proportion of resistant *E*. *coli* samples resistant to ≥1 antimicrobials are presented for all sample types by age, sex, location type, season, and year categories at the sample level in Table 2.

**Table 1:** Percentage (95% CI) of *E*. *coli* positive samples that demonstrate resistance by number of antimicrobial drugs and antimicrobial classes in raccoon fecal, paw, soil, and dumpster samples by location in southern Ontario.

Number of Antimicrobial Drugs	Total	Conservation Area				Swine Farm			
	All Samples [*n* = 2752] ^a^	Feces [*n* = 655]	Soil [*n* = 637]	Paw [*n* = 179]	Dumpster [*n* = 29]	Feces [*n* = 389]	Soil [*n* = 623]	Paw [*n* = 186]	Manure Pit [*n* = 54]
1	4.6 (3.8–5.4)	4.3 (2.8–6.1)	3.8 (2.4–5.6)	3.9 (1.6–7.9)	6.4 (0.8–22.8)	2.3 (1.1–4.3)	6.3 (4.5–8.4)	2.7 (0.9–6.2)	22.2 (12.0–35.6)
2	1.8 (1.3–2.3)	0.6 (0.2–1.6)	0.8 (0.2–2.0)	1.7 (0.3–4.8)	0 (0–11.9) ^b^	2.0 (0.9–4.0)	2.1 (1.1–3.5)	0 (0–0.02) ^b^	24.1 (13.5–37.6)
3–12	3.0 (2.4–3.7)	2.9 (1.8–4.5)	2.7 (1.6–4.2)	3.9 (1.6–7.9)	6.9 (0.8–22.8)	2.3 (1.1–4.3)	3.4 (2.1–5.1)	0.5 (0.01–3.0)	11.1 (4.2–22.6)
**Number of Antimicrobial Drug Classes**									
1	5.6 (4.8–6.6)	5.0 (3.5–7.0)	5.3 (3.7–7.4)	6.1 (3.1–10.7)	6.9 (0.8–22.8)	2.8 (1.4–5.0)	7.4 (5.4–9.7)	2.7 (0.9–6.2)	22.2 (12.0–35.6)
2	1.8 (1.4–2.4)	1.1 (0.4–2.2)	0.9 (3.5–2.0)	2.2 (0.6–5.6)	0 (0–11.9) ^b^	2.0 (0.9–4.0)	2.2 (1.2–3.7)	0 (0–0.02) ^b^	24.1 (13.5–37.6)
3	0.8 (0.5–1.2)	0.6 (0.2–1.6)	0.8 (0.3–1.8)	1.1 (1.4–4.0)	3.4 (0.1–17.8)	0.8 (0.2–2.2)	0.8 (0.3–1.9)	0.5 (0.01–3.0)	5.5 (1.2–15.4)
4	0.7 (0.0–1.1)	0.6 (0.2–1.6)	0.5 (0.1–1.4)	0 (0–0.02) ^b^	0 (0–11.9) ^b^	0.8 (0.2–2.2)	1.1 (0.4–2.3)	0 (0–0.02) ^b^	3.7 (0.4–12.7)
5	0.1 (0.04–0.4)	0 (0–0.006) ^b^	0 (0–0.006) ^b^	0 (0–0.02) ^b^	3.4 (0.1–17.8)	0.2 (0.01–1.4)	0.2 (0.004–0.9) ^b^	0 (0–0.02) ^b^	1.9 (0.05–9.9)
6	0.1 (0.04–0.4)	0.4 (0.1–1.3)	0 (0–0.006) ^b^	0 (0–0.02) ^b^	0 (0–11.9) ^b^	0 (0–0.009) ^b^	0 (0–0.006) ^b^	0 (0–0.02) ^b^	0 (0–0.7) ^b^
**Total Reduced Susceptibility to ≥ 1 Antimicrobial**	**9.3**	**7.8**	**7.5**	**9.5**	**13.8**	**6.7**	**11.7**	**3.2**	**57.4**
	**(8.2–10.4)**	**(5.9–10.1)**	**(5.6–9.9)**	**(5.6–14.8)**	**(3.9–31.7)**	**(4.4–9.6)**	**(9.3–14.5)**	**(1.2–6.9)**	**(43.2–70.8)**
**Total Susceptible**	**90.7**	**92.2**	**92.5**	**90.5**	**86.2**	**93.3**	**88.3**	**96.8**	**40.7**
	**(89.6–91.8)**	**(89.9–94.1)**	**(90.1–94.4)**	**(85.2–94.4)**	**(68.3–96.1)**	**(90.4–95.6)**	**(85.5–90.7)**	**(93.1–98.8)**	**(27.6–55.0)**

^a^ *n* = total number of *E*. *coli* positive samples.

^b^ One-sided, 97.5% confidence interval.

**Table 2:** Percentage (95% CI) of raccoon fecal, raccoon paw, soil, manure pit, and dumpster samples testing positive for *E*. *coli* and having antimicrobial resistance (AMR) to ≥ 1 antimicrobial, by age, sex, location type, season, and year (where applicable) in Ontario from May–November 2011–2013.

Predictor	Category	% with AMR (95% CI) ^a^	% with AMR (95% CI)	% with AMR (95% CI)	% with AMR (95% CI)	% with AMR (95% CI)
		[n]	[n]	[n]	[n]	[n]
		Feces	Soil	Paws	Manure Pit	Dumpster
		[*n* = 1044] ^b^	[*n* = 1260]	[*n* = 365]	[*n* = 54]	[*n* = 29]
Age ^c^	Adult	8.2 (6.3**–**10.4)	— ^d^	5.6 (3.3**–**8.8)	—	—
		[723]		[305]		
	Juvenile	5.6 (3.4**–**8.8)	—	10.0 (3.8**–**20.5)	—	—
		[319]		[60]		
Sex ^c^	Female	7.3 (5.2**–**9.8)	—	6.7 (3.6**–**11.1)	—	—
		[551]		[195]		
	Male	7.5 (5.4**–**10.2)	—	5.9 (2.9**–**10.6)	—	—
		[492]		[170]		
Location type	Swine Farm	6.7 (4.4**–**9.6)	11.7 (9.3**–**14.5)	3.2 (1.2**–**6.9)	57.4 (43.2**–**70.8)	—
		[389]	[623]	[186]	[54]	
	Conservation	7.8 (5.9**–**10.1)	7.5 (5.6**–**9.9)	9.5 (5.6**–**14.8)	—	13.8 (3.9**–**31.7)
	Area	[655]	[637]	[179]		[29]
Season	May to July	7.6 (5.6**–**10.1)	9.7 (7.3**–**12.6)	6.1 (3.3**–**1.0)	55.0 (31.5**–**76.9)	9.1 (0.2**–**41.3)
		[564]	[515]	[231]	[20]	[11]
	Aug. to Nov.	7.1 (5.0**–**9.8)	9.5 (7.5**–**11.9)	6.7 (3.1**–**12.4)	58.8 (40.7**–**75.4)	16.7 (3.6**–**41.4)
		[480]	[745]	[134]	[34]	[18]
Year	2011 ^e^	11.6 (8.3**–**15.5)	14.4 (11.4**–**17.8)	—	64.7 (38.3**–**85.8)	—
		[329]	[494]		[17]	
	2012	5.0 (3.1**–**7.5)	5.8 (3.5**–**9.0)	6.0 (4.0**–**9.3)	42.9 (17.7**–**71.1)	—
		[421]	[310]	[365]	[14]	
	2013	6.1 (3.7**–**9.5)	7.5 (5.2**–**10.5)	—	60.9 (38.5**–**80.3)	12.9 (3.6**–**29.8)
		[294]	[424]		[23]	[31]

^a^ CI = confidence interval.

^b^ *n* = total number of *E*. *coli* positive samples.

^c^ Age was unknown for 2 raccoons and sex was unknown for 1 raccoon fecal sample.

^d^ The dash indicates not applicable.

^e^ Proportion is higher in 2011 than other years because multiple isolates were tested/sample in 2011 in contrast to other years. This autocorrelation was taken into account in the statistical analysis by including a random effect at the sample level.

### Univariable Analyses

The associations between resistant *E*. *coli* isolates and the explanatory variables for raccoon fecal, paw, soil, and manure pit samples are presented in S1 Table. The occurrence of resistant *E*. *coli* isolates was significantly associated with location type for paw and soil samples. Resistant *E*. *coli* isolates were significantly less likely to be detected in raccoon paw samples from swine farms than conservation areas; however, resistant *E*. *coli* isolates were more likely to be detected in soil samples from swine farms than conservation areas. The occurrence of resistant *E*. *coli* isolates was also significantly associated with mean temperature over 14 days prior to sample collection for paw samples; higher mean temperatures 14 days prior to sample collection increased the predicted probability of resistant *E*. *coli* isolates being detected on the paws. There were no associations between the occurrence of resistant *E*. *coli* isolates and any of the explanatory variables in the models for raccoon fecal or manure pit sample isolates.

Although the 95% confidence intervals for variance at the sample and animal levels for raccoon fecal and paw samples, respectively, were large and model fit improved with their exclusion, the random effects at these levels were not removed in the models because: 1) their exclusion resulted in little to no change in the coefficients in the model; and 2) the confidence intervals were believed to be large due to small effective sample sizes. In addition, few animals were recaptured repeatedly in the paw sample analysis and 1–3 isolates were isolated in the raccoon fecal analysis, but during only one year. All of these factors could have resulted in the large confidence intervals we detected at the animal and sample levels.

No multivariable main effects models are presented for any of the sample types because none of the other potential variables were statistically significant or acted as confounders. When a main effects model was created for paw samples, location type was the only term that was significant, and sum of rainfall over 14 days prior to sample collection was no longer significant when included in the model with location type (OR = 0.90; 95% CI; 0.80–1.01; P = 0.080).

### Comparing Sample Types

Resistant *E*. *coli* isolates were more likely to be detected in manure pit samples from swine farms than dumpster samples from conservation areas (S2 Table). There were no significant differences in the proportion of resistant *E*. *coli* isolates between fecal and paw samples collected from the same individual (S2 Table). We analyzed raccoon and fecal samples from swine farms and conservation areas separately because the prevalence of resistant *E*. *coli* isolates in soil samples was significantly different between swine farms and conservation areas. For samples from swine farms, resistant *E*. *coli* isolates were detected more frequently in manure pit than in raccoon fecal and soil samples and more frequently in soil samples than raccoon fecal samples (S2 Table). For samples from conservation areas, there were no significant differences in the proportion of resistant *E*. *coli* isolates between soil samples and raccoon fecal samples, between dumpster samples and raccoon fecal samples, or between dumpster samples and soil samples (S2 Table).

### Diagnostics for Residual Analyses

There were no outlying observations associated with recording errors in any of the significant models. Although the BLUPs were not normally distributed, the random effects were included in the models if: 1) model fit was improved based on the reduction in AIC and BIC when these effects were included; or 2) if excluding them resulted in little to no change to the coefficients in the model.

### Antimicrobial Resistance Phenotypes

The proportions of *E*. *coli* isolates resistant to individual antimicrobials are presented at the sample level for raccoon fecal, raccoon paw, soil, and manure pit samples in S3 Table. Of the antimicrobials of highest importance to human medicine [45], only resistance to AMC and CIP was found in environmental and raccoon samples in both habitat types (S3 Table).

Overall, resistance to TCY, AMP, FOX, STR, and SOX were the top five antimicrobials for which resistance was detected in raccoon fecal and soil samples; resistance to TCY was most common in all sample types overall (S3 Table). For raccoon fecal and soil samples, resistance to FOX was less likely to be detected in *E*. *coli* isolates in 2012 and 2013 than in 2011 (S4 Table). Resistance to STR and TCY was more likely to be detected in *E*. *coli* isolates from soil samples on swine farms than conservation areas (S4 Table).

### Resistance Phenotypic Patterns

Resistance to TCY alone was the most common phenotypic resistance pattern detected and also was the only phenotypic pattern that occurred in all sample types. The second most common phenotypic pattern detected was resistance to FOX alone for raccoon fecal and soil samples and resistance to STR-TCY for manure pit samples. Resistance to CHL alone was not found in manure pit but was found in dumpster samples and was the only phenotypic pattern that was found in raccoon paw, fecal, and soil samples on both conservation areas and swine farms. Sixteen phenotypic patterns consisting of resistance to ≥ 1 antimicrobials were unique to swine farms, 37 were unique to conservation areas, and 20 were common to both location types.

Resistance to ≥ 2 antimicrobials was detected in 4.8% of isolates (Table 1). Seventeen phenotypic patterns consisting of ≥ 2 antimicrobials were detected in samples from both location types, including resistance to GEN-STR-SOX-TCY, AMP-CHL-SOX-TCY, and AMC-AMP-FOX-TCY. However, some multidrug patterns were detected in sample types from only one location type. For example, resistance to AMC-AMP-FOX-TIO-CRO and to GEN-STR-SOX-TCY were detected in *E*. *coli* isolates from raccoon fecal, paw and soil samples and raccoon paw and soil samples from conservation areas, respectively, but were not detected in any of the *E*. *coli* isolates from swine farm samples. In addition, resistance to CHL-STR-SOX-SXT-TCY was detected in *E*. *coli* isolates from manure pit, raccoon fecal, and soil samples from swine farms but was not detected in any *E*. *coli* isolates from samples from conservation areas.

One isolate from a raccoon fecal sample from a conservation area was resistant to 12 antimicrobials (AMC-AMP-FOX-TIO-CRO-CHL-CIP-GEN-NAL-STR-SOX-TCY). Resistance to up to 7 and 10 antimicrobials was detected in *E*. *coli* isolates from swine farms in soil and raccoon fecal samples, respectively. Resistance to up to 6 antimicrobials was detected in *E*. *coli* isolates from dumpster, manure pit, and paw samples from conservation areas, whereas resistance to up to 4 antimicrobials was detected in *E*. *coli* isolates from raccoon paw samples from swine farms.

### Antimicrobial Resistance Genes

The proportion of AMR genes detected in resistant *E*. *coli* isolates is presented in S5 Table for all sample types at the sample level. At the isolate level, resistance genes were detected in 71.0% (196/276) of all phenotypically resistant isolates tested; however, excluding those with intermediate susceptibility, resistant genes were detected in 90.0% (194/218) of resistant isolates. Extended-spectrum beta-lactamase (ESBL) and extended-spectrum cephamycinase producing *E*. *coli*, indicated by the *bla*_CTX-M_ and *bla*_CMY-2_ genes, respectively, were detected in 6 raccoon fecal, 2 soil, and 2 paw samples from conservation areas, but only 1 raccoon fecal sample from swine farms (S5 Table). The *bla*_CMY-2_ gene as well as the *sul1*, *sul2*, *tet*(A), *strA/B*, *aadA*, and *floR* genes were detected in the *E*. *coli* isolate from the raccoon fecal sample from the conservation areas that had resistance to 12 antimicrobials. The *bla*_CTX-M_ gene was found in an isolate with the resistance pattern of AMP-TIO-CRO-TCY from 1 raccoon fecal sample from a conservation area, but was not detected from any other sample types and was completely absent from swine farms (S5 Table); DNA sequencing showed that it was a *bla*_CTX-M-27_ variant.

## Discussion

We found no difference in the prevalence of resistant *E*. *coli* in raccoon fecal samples from swine farms and conservation areas. This is consistent with what has been found when comparing raccoon fecal samples in rural and urban areas in Ontario [32], but in contrast to Allen et al. [10] who found a higher prevalence of resistant *E*. *coli* isolates in mice living on swine farms (48%) than residential areas in Ontario (5%; [10]). The similar prevalence of resistant *E*. *coli* isolates from raccoon feces between conservation areas and swine farms may be attributed to the large home ranges of raccoons that can span up to 4 km^2^ in Ontario [44]. Antimicrobial resistance phenotypes that were absent from the swine farm environment were found in raccoon fecal samples from swine farms and raccoon fecal and soil samples from conservation areas suggesting that raccoons may be acquiring AMR from sources other than the environment in which they were trapped.

The prevalence of resistant *E*. *coli* in raccoon fecal samples in this study (7%; 95% CI, 6–9) is lower than what has been detected in *E*. *coli* isolates from free-ranging raccoons sampled from zoo sites (42%; 95% CI, 34–51; [32]), but is similar to what has been detected in wild mice living in natural habitats (5%; 95% CI, 0.1–23) and residential areas (9%; 95% CI, 3–18) in Ontario [10]. The higher overall prevalence of resistant *E*. *coli* isolates in raccoons reported by Jardine et al. [32] might be attributed to raccoons at the zoo having more direct contact with medicated feed/water or waste in the environment where treated zoo animals were housed [32]. Because all of the swine in our study were housed indoors, raccoons in our study did not come into direct contact with the environment swine were housed or any fresh swine feces. Because raccoons have large home-ranges, it is also possible that raccoons captured in the swine farm environment did not frequently forage in the swine farm environment, which may have decreased their exposure.

Small rodents have much smaller home-ranges than raccoons, so antimicrobial resistant bacteria found in mice likely reflects what is in the immediate environment. For example, we found a higher prevalence of resistant *E*. *coli* and *E*. *coli* isolates resistant to TCY and STR from soil samples on swine farms compared to conservation areas, which is consistent with what has been found in wild mice [10, 13], but in contrast to what we found in raccoons in this study. Although raccoons may also act as sentinels of AMR in the environment, it likely is at larger spatial scales than mice or environmental samples collected from a few locations.

The prevalence of resistant *E*. *coli* from raccoon fecal samples did not differ between swine farms and conservation areas, but we found different resistance phenotypes and genotypes in each habitat type. For example, we detected a wider variety of resistant *E*. *coli* phenotypes from conservation areas than swine farms, which is similar to the results of another environmental study that found a higher diversity of AMR genes in municipal human waste effluent than from livestock environments [46]. In addition, the prevalence of resistance to TIO, CRO, NAL, and GEN in general is low in *E*. *coli* isolates from Canadian beef and swine [47], and our results from environmental samples from swine farms agreed with those results. Because resistance to these antimicrobials was found in soil samples on conservation areas, resistance to these antimicrobials may be associated with sources other than swine or cattle manure.

Differences in sources of antimicrobial resistant bacteria between swine farms and conservation areas is further suggested by the ESBL and cephamycinase producing *E*. *coli* genes (*bla*_*CTX-M*_ and *bla*_*CMY-2*_, respectively) being nearly absent in all samples from swine farms, but being found in raccoon paw, and soil samples from conservation areas. Although the *bla*_*CTX-M*_ genes have been found to be associated with humans, livestock, wildlife and environmental samples [31], we detected a *bla*_CTX-M-27_ variant in a raccoon fecal sample from a conservation area. This supports the hypothesis of a human source in our study because this subtype is frequently detected in *E*. *coli* associated with both hospital and community acquired extraintestinal *E*. *coli* infections in humans in this region of Ontario [48].

Surprisingly, raccoons in conservation areas had a higher prevalence of resistant *E*. *coli* on their paws than raccoons from swine farms. Detection of resistant *E*. *coli* isolates from dumpster samples suggests that human refuse, including raw meat and other food waste, could be possible sources of resistant *E*. *coli* for raccoon paws in conservation areas. Unfortunately, it is difficult to draw any conclusions about the role of dumpsters as a source of resistant bacteria because the effective sample size of dumpster samples was small and we only sampled them during one year.

Pet dog feces may be another potential source of antimicrobial resistant bacteria [49] to raccoons and the soil in conservation areas. The prevalence of resistant *E*. *coli* isolates in raccoon fecal (8%; 95% CI, 6–10) and soil samples (8%; 95% CI, 6–10) from conservation areas was slightly lower to what was found in pet dogs visiting dog parks in southwestern Ontario in 2009 (11%; 95% CI, 9–14; [50]). However, resistance to CIP or NAL was not detected in *E*. *coli* isolates from any dog fecal samples in contrast to raccoon and soil samples from conservation areas in this study, suggesting that exposure to *E*. *coli* with these resistance phenotypes in this habitat type are from sources other than dogs.

Although raccoons commonly use and feed in aquatic habitats [51] and may be exposed to resistant bacteria from water [52], sediment, or biofilms [53] from contaminated rivers, tributaries, and streams, we did not find any significant associations between the prevalence of resistant *E*. *coli* and year, season, and rainfall in any of the sample types, which is in contrast to what has been found with the prevalence of *Salmonella* in raccoon fecal, soil and/or manure pit samples [36]. These differences might be related to the biology of these bacteria or by the small effective sample sizes for resistant *E*. *coli*, which may have limited study power. In a study of an agricultural watershed in British Columbia, seasonality had no relationship with resistance frequency of *E*. *coli* isolates in aquatic samples; however, many physical and chemical factors (e.g., water depth, nutrient concentrations, temperature, dissolved oxygen, and salinity) had statistically significant associations with frequency of resistance to NAL, STR, AMP, and TCY [54].

The prevalence of resistant *E*. *coli* isolates was low in this study. Much higher levels of resistance have been found in retail pork (36–46%), beef (30–37%), and chicken (25–32%) samples in Ontario [47, 54, 55] than raccoon fecal samples collected during the same time period in this study (5–11%). Although raccoons have the potential to directly disseminate resistant bacteria over larger distances than small mammals, previous studies have shown that raccoons are unlikely to carry specific *E*. *coli* serotypes over long periods of time [32]. This suggests that raccoons are likely acquiring resistant bacteria from their environment rather than maintaining them.

## Conclusion

The prevalence of resistant *E*. *coli* isolates in raccoon fecal samples did not differ between swine farms and conservation areas in southern Ontario, but the prevalence of resistant *E*. *coli* isolates from soil samples was higher on swine farms than conservation areas. Although the source of environmental resistance is unclear, we found different resistance phenotypes and genotypes in each location type, which suggests that the exposure differs between location types. Because raccoons have large home ranges, AMR detected in their feces most likely represent resistance found in the environment on a larger scale than environmental samples collected in areas where they were trapped. Raccoons can be used as sentinels for detecting environmental AMR. However, our results strongly suggest that caution should be used when categorically classifying location types in AMR studies as being natural if there is any level of human use in the area, in particular for environmental and wildlife samples. For wildlife species with larger home-ranges, such as raccoons, future studies simultaneously radio-collaring individual animals and/or analyzing diet are needed to better understand exposure in relation to carriage of antimicrobial resistant bacteria.

## Supporting Information

S1 Table Univariable logistic regression models showing associations between the occurrence of *E*. *coli* isolates resistant to ≥ 1 antimicrobial in raccoon fecal and paw, soil, and manure pit samples with respect to raccoon age and sex, location type, and year (2011–2013), if applicable, and season, sum of rainfall, and mean temperature in Ontario, Canada. (DOCX)

S2 Table Univariable logistic regression models for the association of *E*. *coli* isolates with resistance to ≥ 1 antimicrobial and sample type. (DOCX)

S3 Table Percentage (95% CI) of *E*. *coli* isolates resistant to individual antimicrobials from all sample types overall and on conservation areas and swine farms in southern Ontario. (DOCX)

S4 Table Multi-level univariable models for the most common antimicrobial drugs resistance was detected in *E*. *coli* isolates from raccoon fecal and soil samples. (DOCX)

S5 Table Percentage (95% CI) of antimicrobial resistance genes in resistant *E*. *coli* isolates for all sample types overall and on conservation and swine farms in southern Ontario. (DOCX)
