# Supplementary material for: Epidemiology of Antimicrobial Resistance in Escherichia coli Isolates from Raccoons (Procyon lotor) and the Environment on Swine Farms and Conservation Areas in Southern Ontario
Source: PLoS One. 2016 Nov 9;11(11):e0165303. doi: 10.1371/journal.pone.0165303 (PMC5102455; doi:10.1371/journal.pone.0165303)
Supplement: S1 Table — (DOCX) [file pone.0165303.s001.docx]

**Table S1. Univariable logistic regression models showing associations between the occurrence of *E. coli* isolates resistant to ≥ 1 antimicrobial in raccoon fecal and paw, soil, and manure pit samples with respect to raccoon age and sex, location type, and year (2011**–**2013), if applicable, and season, sum of rainfall, and mean temperature in Ontario, Canada.**

| **Univariable models for resistant *E. coli* isolates according to sample type ^a^** | | | | | | | | |
| --- | --- | --- | --- | --- | --- | --- | --- | --- |
| **Raccoon feces (*n*=1570) ^b^** | | | | | **Variance [VPC] ^c^**  **(95% CI) ^d^** | | | |
| Predictor | Category | Odds  Ratio | 95% CI | *P* | Site-level | Animal-level | Sample-level | Isolate- level |
| Sex | Male (Female REF) ^e^ | 1.15 | 0.68–1.95 | 0.593 | 0.06 [1.4] | 0.95 [21.8] | 0.06 [1.4] | [75.4] |
|  |  |  |  |  | (0.001–2.61) | (0.30–3.01) | (4.3 x 10^-13^–8.2 x 10^9^) |  |
| Age | Juvenile (Adult REF) | 0.57 | 0.32–1.04 | 0.070 | 0.06 [1.4] | 0.82 [19.6] | 0.08 [0.19] | [78.7] |
|  |  |  |  |  | (0.001–2.20) | (0.23–2.97) | (4.0 x 10^-10^–1.6 x 10^7^) |  |
| Location Type | Swine farm | 1.01 | 0.54–1.90 | 0.978 | 0.06 [1.4] | 0.93 [21.4] | 0.06 [1.4] | [75.8] |
|  | (Conservation Area REF) |  |  |  | (0.001–2.80) | (0.29–3.00) | (3.6 x 10^-12^–1.2 x 10^9^) |  |
| Season | Aug. to Nov. | 0.73 | 0.45–1.20 | 0.220 | 0.04 [0.9] | 0.91 [21.4] | 0.01 [0.2] | [77.4] |
|  | (May to July REF) |  |  |  | (3.7 x 10^3^–5.0) | (0.28–2.96) | (1.1 x 10^-66^–9.5 x 10^61^) |  |
| Year ^f^ | 2012 (2011 REF) | 0.96 | 0.51–1.81 | 0.905 | 0.06 [1.4] | 0.92 [21.2] | 0.07 [1.6] | [75.8] |
|  |  |  |  |  | (0.001–2.87) | (0.28–2.96) | (1.3 x 10^-11^–3.6 x 10^8^) |  |
|  | 2013 (2011 REF) | 1.28 | 0.67–2.43 | 0.460 | 0.06 [1.4] | 0.92 [21.2] | 0.07 [1.6] | [75.8] |
|  |  |  |  |  | (0.001–2.87) | (0.28–2.96) | (1.3 x 10^-11^–3.6 x 10^8^) |  |
| Sum of rainfall | 30 days | 1.00 | 0.10–1.00 | 0.692 | 0.06 [1.4] | 0.93 [21.3] | 0.09 [2.0] | [75.3] |
|  |  |  |  |  | (0.002–2.58) | (0.29–3.01) | (6.28 x 10^-9^–1.4 x 10^6^) |  |
| Sum of rainfall | 14 days | 1.00 | 0.99–1.01 | 0.839 | 0.06 [1.4] | 0.93 [21.3] | 0.08 [1.8] | [75.4] |
|  |  |  |  |  | (0.001–2.71) | (0.29–3.00) | (4.3 x 10^-10^–1.6 x 10^7^) |  |
| Sum of rainfall | 3 days | 1.01 | 0.99–1.03 | 0.327 | 0.07 [1.6] | 0.90 [20.7] | 0.09 [2.1] | [75.6] |
|  |  |  |  |  | (0.002–2.17) | (0.27–2.99) | (2.5 x 10^-9^–3.1 x 10^6^) |  |
| Mean temperature | 30 days | 0.99 | 0.92–1.06 | 0.781 | 0.06 [1.4] | 0.93 [21.4] | 0.07 [1.6] | [75.6] |
|  |  |  |  |  | (0.001–2.82) | (0.29–2.99) | (3.4 x 10^-11^–1.5 x 10^8^) |  |
| Mean temperature | 14 days | 1.00 | 0.94–1.06 | 0.925 | 0.06 [1.4] | 0.93 [21.4] | 0.07 [1.6] | [75.6] |
|  |  |  |  |  | (0.001–2.82) | (0.29–3.00) | (7.9 x 10^-12^–5.7 x 10^8^) |  |
| Mean temperature | 3 days | 1.03 | 0.97–1.08 | 0.354 | 0.06 [1.4] | 0.94 [21.8] | 0.03 [0.7] | [76.1] |
|  |  |  |  |  | (0.001–2.65) | (0.29–3.01) | (3.4 x10^-26^–2.3 x 10^22^) |  |
| **Raccoon Paws (*n*=365) ^b^** | | | | | **Variance [VPC] ^c^**  **(95% CI) ^d^** | | |  |
| Predictor | Category | Odds Ratio | 95% CI | *P* | Site-level | Animal-level | Sample/isolate-level |  |
| Sex | Male (Female REF) ^e^ | 0.92 | 0.38–2.20 | 0.843 | 0.15 [4.2] | 0.11 [3.1] | [92.7] |  |
|  |  |  |  |  | (0.005–4.64) | (1.3 x 10^-10^–9.4 x 10^7^) |  |  |
| Age | Juvenile (Adult REF) | 1.74 | 0.62–4.95 | 0.294 | 0.09 [2.6] | 0.06 [1.7] | [95.6] |  |
|  |  |  |  |  | 5.9 x 10^-4^–14.71) | (4.7 x 10^-17^–7.6 x 10^13^) |  |  |
| Location type | Swine farm | **0.32** | **0.12–0.83** | **0.019** | **—  ^g^** | **0.04 [0.90]** | **[99.1]** |  |
|  | (Conservation area REF) |  |  |  |  | (2.5 x 10^-23^–6.3 x 10^23^) |  |  |
| Season | Aug. to Nov. | 1.34 | 0.56–3.20 | 0.510 | 0.13 [3.8] | 0.01 [0.3] | [95.9] |  |
|  | (May to July REF) |  |  |  | (0.003–5.49) | (1.1 x 10^-108^–6.9 x 10^103^) |  |  |
| Sum of rainfall | 30 days | 1.01 | 0.10–1.03 | 0.068 | 0.09 [2.7] | —  ^g^ | [97.3] |  |
|  |  |  |  |  | (2.2 x 10^-10^–5.3 x 10^7^) |  |  |  |
| Sum of rainfall | 14 days | 1.00 | 0.99–1.02 | 0.233 | 0.18 [5.2] | —  ^g^ | [94.8] |  |
|  |  |  |  |  | (0.008–4.34) |  |  |  |
| Sum of rainfall | 3 days | 1.01 | 0.97–1.06 | 0.661 | 0.19 [5.3] | 0.13 [3.6] | [91.1] |  |
|  |  |  |  |  | (0.008–4.21) | (3.4 x 10^-9^–5.0 x 10^6^) |  |  |
| Mean temperature | 30 days | 0.90 | 0.79–1.03 | 0.115 | 0.15 [4.3] | 0.07 [0.6] | [95.1] |  |
|  |  |  |  |  | (0.005–4.32) | (1.2 x 10^-17^–4.4 x 10^12^) |  |  |
| Mean temperature | 14 days | **0.89** | **0.79–1.00** | **0.047** | **0.15 [4.3]** | **0.02 [0.6]** | **[95.1]** |  |
|  |  |  |  |  | **(0.004**–**4.75)** | **(6.9 x 10^-62^–3.7 x 10^57^)** |  |  |
| Mean temperature | 3 days | 1.00 | 0.90–1.11 | 0.969 | 0.15 [4.2] | 0.10 [2.8] | [92.9] |  |
|  |  |  |  |  | (0.006–4.29) | (3.1 x 10^-11^–3.5 x 10^8^) |  |  |
| **Soil (*n*=2000) ^b^** | | | | | **Variance [VPC] ^c^**  **(95% CI) ^d^** | | |  |
| Predictor | Category | Odds  Ratio | 95% CI | *P* | Site-level | Sample-level | Isolate-level |  |
| Location type | Swine farm | **1.80** | **1.16–2.80** | **0.009** | **—  ^g^** | **1.73 [34.5]** | **[65.5]** |  |
|  | (Conservation area REF) ^e^ |  |  |  |  | **(0.64**–**4.68)** |  |  |
| Season | Aug. to Nov. | 0.96 | 0.62–1.49 | 0.863 | 0.06 [1.2] | 1.80 [35.0] | [63.9] |  |
|  | (May to July REF) |  |  |  | (0.003–1.15) | (0.67–4.80) |  |  |
| Year ^f^ | 2012 (2011 REF) | 0.84 | 0.45–1.57 | 0.588 | 0.06 [1.2] | 1.80 [35.0] | [63.9] |  |
|  |  |  |  |  | (0.003–1.27) | (0.67–4.86) |  |  |
|  | 2013 (2011 REF) | 1.04 | 0.62–1.75 | 0.888 | 0.06 [1.2] | 1.80 [35.0] | [63.9] |  |
|  |  |  |  |  | (0.003–1.27) | (0.67–4.86) |  |  |
| Sum of rainfall | 30 days | 1.00 | 0.10–1.01 | 0.567 | 0.06 [1.2] | 1.80 [35.0] | [63.9] |  |
|  |  |  |  |  | (0.003–1.13) | (0.67–4.80) |  |  |
| Sum of rainfall | 14 days | 1.00 | 1.00–1.01 | 0.450 | 0.05 [1.0] | 1.80 [35.0] | [64.0] |  |
|  |  |  |  |  | (0.002–1.31) | (0.68–4.78) |  |  |
| Sum of rainfall | 3 days | 1.01 | 1.00–1.03 | 0.051 | 0.02 [0.39] | 1.84 [35.7] | [63.9] |  |
|  |  |  |  |  | (1.6 x 10^-5^–25.14) | (0.70–4.82) |  |  |
| Mean temperature | 30 days | 1.00 | 1.00–1.06 | 0.920 | 0.06 [1.2] | 1.78 [34.7] | [64.1] |  |
|  |  |  |  |  | (0.003–1.16) | (0.67–4.77) |  |  |
| Mean temperature | 14 days | 1.01 | 0.96–1.06 | 0.760 | 0.06 [1.2] | 1.80 [35.0] | [63.9] |  |
|  |  |  |  |  | (0.003–1.15) | (0.68–4.81) |  |  |
| Mean temperature | 3 days | 1.01 | 0.97–1.05 | 0.696 | 0.06 [1.2] | 1.82 [35.2] | [63.6] |  |
|  |  |  |  |  | (0.004–1.10) | (0.68–4.83) |  |  |
| **Manure pit (*n*=86) ^b^** | | | | | **Variance [VPC] ^c^**  **(95% CI) ^d^** | | |  |
| Predictor | Category | Odds Ratio | 95% CI | *P* | Site-level | Sample-level | Isolate-level |  |
| Season | Aug. to Nov. | 0.81 | 0.18–3.68 | 0.787 | 0.13 [2.2] | 2.68 [41.0] | [56.7] |  |
|  | May to July (REF) ^e^ |  |  |  | (7.8 x 10^-5^–230) | (0.40–17.81) |  |  |
| Year ^f^ | 2012 (2011 REF) | 0.93 | 0.13–6.91 | 0.946 | 0.17 [2.8] | 2.60 [42.9] | [54.3] |  |
|  |  |  |  |  | 3.0 x 10^-4^–98.69) | (0.39–17.49) |  |  |
|  | 2013 (2011 REF) | 3.01 | 0.53–17.0 | 0.211 | 0.17 [2.8] | 2.60 [42.9] | [54.3] |  |
|  |  |  |  |  | (3.0 x 10^-4^–98.69) | (0.39–17.49) |  |  |
| Sum of rainfall | 30 days | 1.00 | 0.98–1.01 | 0.933 | 0.14 [2.3] | 2.67 [43.8] | [53.9] |  |
|  |  |  |  |  | (9.0 x 10^-5^–210) | (0.39–17.91) |  |  |
| Sum of rainfall | 14 days | 0.98 | 0.96–1.01 | 0.157 | 0.06 [1.1] | 1.93 [36.6] | [62.3] |  |
|  |  |  |  |  | (6.2 x 10^-8^–5.1 x 10^8^) | (0.22–16.95) |  |  |
| Sum of rainfall | 3 days | 0.99 | 0.93–1.06 | 0.798 | 0.16 [2.6] | 2.69 [43.8] | [53.6] |  |
|  |  |  |  |  | (2.0 x 10^-4^–120) | (0.41–17.81) |  |  |
| Mean temperature | 30 days | 0.90 | 0.72–1.11 | 0.304 | 0.12 [1.9] | 2.92 [46.2] | [51.9] |  |
|  |  |  |  |  | (3.0 x 10^-5^–580) | (0.48–17.95) |  |  |
| Mean temperature | 14 days | 0.91 | 0.75–1.10 | 0.344 | 0.11 [1.8] | 2.85 [45.6] | [52.6] |  |
|  |  |  |  |  | (1.0 x 10^-5^–990) | (0.46–17.88) |  |  |
| Mean temperature | 3 days | 0.98 | 0.86–1.11 | 0.734 | 0.14 [2.3] | 2.79 [44.9] | [52.9] |  |
|  |  |  |  |  | (7.0 x 10^-5^–260) | (0.43–18.12) |  |  |

^a^ Significant differences are highlighted in bold.

^b^ *n* = number of isolates.

^c^ Variance partition coefficient.

^d^ CI = confidence interval.

^e^ REF is the referent group.

^f^ Wald’s χ^2^ test for year was *P* = 0.692 for raccoon fecal samples, *P* = 0.822 for soil samples, and *P* = 0.395 for manure pit samples.

^g^ Random effect was not included because it did not improve the fit of the model, it explained only a

small amount of the variance (6.4 x 10^-33^ to 7.7 x 10^-29^ ), and its removal had little to no impact on the

coefficients in the models.
