# Supplementary material for: Epidemiology of Antimicrobial Resistance in Escherichia coli Isolates from Raccoons (Procyon lotor) and the Environment on Swine Farms and Conservation Areas in Southern Ontario
Source: PLoS One. 2016 Nov 9;11(11):e0165303. doi: 10.1371/journal.pone.0165303 (PMC5102455; doi:10.1371/journal.pone.0165303)
Supplement: S2 Table — (DOCX) [file pone.0165303.s002.docx]

**Table S2. Univariable logistic regression models for the association of *E. coli* isolates with resistance to ≥ 1 antimicrobial and sample type.**

| **Univariable models for *E. coli* isolates with resistance to ≥ 1 antimicrobial ^a^** | | | | | | | | |
| --- | --- | --- | --- | --- | --- | --- | --- | --- |
|  |  |  |  |  | **Variance [VPC] ^b^** | | | |
|  |  |  |  |  | **(95% CI) ^c^** | | | |
| **Sample Type Comparison** | ***n* ^d^** | **OR ^e^** | **95% CI** | ***P*** | **Site-level** | **Animal/dumpster/**  **manure pit-level** | **Sample-level** | **Isolate-level** |
| Manure pit vs. dumpster | 115 | **11.74** | **1.51**–**91.0** | **0.018** | — ^f^ | **0.45 [7.6]** | **2.15 [36.6]** | **[55.8]** |
|  |  |  |  |  |  | **(0.30–6.78)** | **(0.26–17.9)** |  |
| Raccoon fecal vs. raccoon paw | 690 | 1.40 | 0.68–2.89 | 0.363 | — ^f^ | 2.37 [41.9] | [58.1] | — ^g^ |
|  |  |  |  |  |  | (0.82–6.81) |  |  |
|  | **Swine Farm** | | |  |  |  |  |  |
| **Sample Type Comparisons** | ***n*** | **OR** | **95% CI** | ***P*** |  |  |  |  |
| Manure pit vs. raccoon fecal | 1618 | **39.69** | **12.03**–**130.91** | **≤ 0.001** | **0.14 [2.5]** | **0.56 [10.2]** | **1.49 [27.2]** | **[60.1]** |
|  |  |  |  |  | **(0.02–1.08)** | **(0.06–4.90)** | **(0.41–5.37)** |  |
| Manure pit vs. soil |  | **21.21** | **7.14**–**63.06** | **≤ 0.001** | **0.14 [2.5]** | **0.56 [10.2]** | **1.49 [27.2]** | **[60.1]** |
|  |  |  |  |  | **(0.02–1.08)** | **(0.06–4.90)** | **(0.41–5.37)** |  |
| Soil vs. raccoon fecal |  | **1.87** | **1.07**–**3.29** | **0.029** | **0.14 [2.5]** | **0.56 [10.2]** | **1.49 [27.2]** | **[60.1]** |
|  |  |  |  |  | **(0.02–1.08)** | **(0.06–4.90)** | **(0.41–5.37)** |  |
|  | **Conservation Area** | | |  |  |  |  |  |
| **Sample Type Comparisons** | ***n*** | **OR** | **95% CI** | ***P*** |  |  |  |  |
| Soil vs. raccoon fecal | 2067 | 0.96 | 0.62–1.50 | 0.869 | — ^f^ | 0.79 [18.6] | 0.16 [3.8] | [77.6] |
|  |  |  |  |  |  | 0.22–2.79 | 2.1 x 10^-5^–1242.3 |  |
| Dumpster vs. raccoon fecal |  | 3.39 | 0.88–13.10 | 0.077 | — ^f^ | 0.79 [18.6] | 0.16 [3.8] | [77..6] |
|  |  |  |  |  |  | 0.22–2.79 | 2.1 x 10^-5^–1242.3 |  |
| Dumpster vs. soil |  | 3.52 | 0.91–13.54 | 0.067 | — ^f^ | 0.79 [18.6] | 0.16 [3.8] | [77.6] |
|  |  |  |  |  |  | 0.22–2.79 | 2.1 x 10^-5^–1242.3 |  |

^a^ Significant differences are highlighted in bold.

^b^ VPC = variance partition coefficient.

^c^ CI = confidence interval.

^d^ *n*= total number of isolates in analysis.

^e^ OR = odds ratio.

^f^ Site explained 5.7 x 10^-31^–3.3 x 10^-18^ of the variance, so it was excluded from the analysis.

^g^ Random effect for isolate was not included because only 1 isolate per sample was collected for *E. coli* samples in this analysis.
