# Supplementary material for: Epidemiology of Antimicrobial Resistance in Escherichia coli Isolates from Raccoons (Procyon lotor) and the Environment on Swine Farms and Conservation Areas in Southern Ontario
Source: PLoS One. 2016 Nov 9;11(11):e0165303. doi: 10.1371/journal.pone.0165303 (PMC5102455; doi:10.1371/journal.pone.0165303)
Supplement: S3 Table — (DOCX) [file pone.0165303.s003.docx]

**TABLE S3. Percentage (95% CI) of *E. coli* isolates resistant to individual antimicrobials from all sample types overall and on conservation areas and swine farms in southern Ontario.**

|  |  | **% (95% CI) Overall ^a, b^** | | | | |  | **% (95% CI) Conservation Area ^a, b^** | | |  | **% (95% CI) Swine Farm ^a, b^** | | |  |
| --- | --- | --- | --- | --- | --- | --- | --- | --- | --- | --- | --- | --- | --- | --- | --- |
|  |  | **Feces** | **Soil** | **Paws** | **Manure pit** | **Dumpster** | **Total** | **Feces** | **Soil** | **Paws** | **Total** | **Feces** | **Soil** | **Paws** | **Total** |
| **Category ^c^** | **Drug ^d^** | [*n* = 1044] ^e^ | [n=1260] | [n=365] | [n=54] | [*n*=29] | [*n*=2752] | [*n*=655] | [*n*=637] | [*n*=179] | [*n*=1471] | [*n*=389] | [*n*=623] | [*n*=186] | [*n*=1198] |
| I | AMC ^f^ | **1.1** | **1.7** | **1.4** | **1.8** | **— ^g^** | **1.4** | **1.2** | **1.9** | **2.8** | **1.7** | **1.0** | **1.6** | **—** | **1.2** |
|  |  | (0.6–2.0) | (1.1–2.6) | (0.4–3.2) | (0.05–9.9) |  | (1.0–2.0) | (0.5–2.4) | 0.1–3.3) | (0.9–6.4) | (1.1–2.5) | (0.3–2.6) | (0.8–2.9) |  | (0.6–2.0) |
| I | CRO | **0.6** | **0.2** | **0.5** | **—** | **—** | **0.4** | **0.8** | **0.4** | **1.1** | **0.6** | **0.3** | **—** | **—** | **0.1** |
|  |  | (0.2–1.2) | (0.02–0.6) | (0.1–2.0) |  |  | (0.2–0.7) | (0.2–1.8) | (0.04–1.1) | (0.1–4.0) | (0.3–1.2) | (0.01–1.4) |  |  | (0.002–0.5) |
| I | CIP ^e^ | **0.5** | **0.4** | **—** | **—** | **—** | **0.4** | **0.6** | **0.5** | **—** | **0.5** | **0.3** | **0.3** | **—** | **0.2** |
|  |  | (0.2–1.1) | (0.1–0.9) |  |  |  | (0.2–0.7) | (0.2–1.6) | (0.1–1.4) |  | (0.2–1.0) | (0.01–1.4) | (0.01–1.4) |  | (0.05–0.7) |
| I | TIO | **0.6** | **0.2** | **0.5** | **—** | **—** | **0.4** | **0.8** | **0.4** | **1.1** | **0.6** | **0.3** | **—** | **—** | **0.1** |
|  |  | (0.2–2.0) | (0.02–0.6) | (0.1–2.0) |  |  | (0.2–0.7) | (0.2–1.8) | (0.04–1.1) | (0.1–4.0) | (0.3–1.2) | (0.01–1.4) |  |  | (0.002–0.5) |
| II | AMP ^f^ | **3.0** | **2.9** | **1.6** | **16.7** | **3.4** | **3.0** | **3.4** | **2.5** | **3.4** | **3.0** | **2.3** | **3.4** | **—** | **2.5** |
|  |  | (2.0–4.2) | (2.1–4.0) | (0.6–3.5) | (7.9–29.3) | (0.1–17.8) | (2.4–3.8) | (2.1–5.0) | (1.4–4.0) | (1.2–7.2) | (2.2–4.0) | (1.1–4.3) | (2.1–5.1) |  | (1.7–3.6) |
| II | FOX ^f^ | **2.4** | **3.1** | **2.2** | **—** | **—** | **2.6** | **3.0** | **2.7** | **2.8** | **2.9** | **1.3** | **3.5** | **1.6** | **2.5** |
|  |  | (1.6–3.5) | (2.2–4.2) | (1.0–4.3) |  |  | (2.1–3.3) | (1.9–4.7) | (1.6–4.2) | (0.9–6.4) | (2.1–3.8) | (0.4–3.0) | (2.2–5.3) | (0.3–4.6) | (1.7–3.6) |
| II | GEN | **0.5** | **0.2** | **0.3** | **—** | **3.4** | **0.3** | **0.6** | **0.5** | **—** | **0.5** | **0.3** | **—** | **0.5** | **0.2** |
|  |  | (0.2–1.1) | (0.05–0.7) | (0.01–1.5) |  | (0.1–17.8) | (0.1–0.6) | (0.2–1.6) | (0.1–1.4) |  | (0.2–1.0) | (0.01–1.4) |  | (0.01–3.0) | (0.02–0.6) |
| II | KAN ^f^ | **0.5** | **0.6** | **0.3** | **1.9** | **—** | **0.6** | **0.4** | **0.8** | **0.6** | **0.6** | **0.5** | **0.3** | **—** | **0.3** |
|  |  | (0.2–1.1) | (0.2–1.1) | (0.01–1.5) | (0.05–9.9) |  | (0.03–1.0) | (0.1–1.3) | (0.3–1.8) | (0.01–3.1) | (0.3–1.2) | (0.1–1.8) | (0.04–1.1) |  | (0.1–0.9) |
| II | NAL | **0.4** | **0.1** | **0.3** | **—** | **—** | **0.2** | **0.6** | **0.2** | **0.6** | **0.4** | **—** | **—** | **—** | **—** |
|  |  | (0.1–1.0) | (0.002–0.4) | (0.01–1.5) |  |  | (0.1–0.5) | (0.2–1.6) | (0.004–0.9) | (0.01–3.1) | (0.1–0.9) |  |  |  |  |
| II | STR | **1.8** | **2.5** | **0.8** | **29.6** | **3.4** | **2.9** | **1.4** | **1.6** | **0.6** | **1.5** | **2.6** | **3.5** | **1.1** | **2.8** |
|  |  | (1.1–2.8) | (1.7–3.6) | (0.2–2.4) | (18.0–43.6) | (0.1–17.8) | (2.3–3.6) | (0.6–2.6) | (0.8–2.9) | (0.01–3.1) | (0.9–2.3) | (1.2–4.7) | (2.2–5.3) | (0.1–3.8) | (2.0–3.9) |
| II | SXT | **1.0** | **1.2** | **1.1** | **1.9** | **3.4** | **1.2** | **0.8** | **1.1** | **2.2** | **1.1** | **1.5** | **1.3** | **—** | **1.2** |
|  |  | (0.5–1.9) | (0.7–2.0) | (0.3–2.8) | (0.05–9.9) | (0.1–17.8) | (0.8–1.6) | (0.2–1.8) | (0.4–2.3) | (0.6–5.6) | (0.6–1.8) | (0.6–3.3) | (0.6–2.5) |  | (0.6–2.0) |
| III | CHL^f^ | **1.2** | **1.0** | **1.6** | **3.7** | **6.9** | **1.3** | **1.2** | **0.5** | **3.4** | **1.1** | **1.5** | **1.6** | **0.5** | **1.3** |
|  |  | (0.7–2.1) | (0.6–1.8) | (0.6–3.5) | (0.4–12.7) | (0.8–22.8) | (0.9–1.8) | (0.5–2.4) | (0.1–1.4) | (1.2–7.2) | (0.6–1.8) | (0.6–3.3) | (0.8–2.9) | (0.01–3.0) | (0.8–2.2) |
| III | SOX | **2.0** | **2.0** | **1.4** | **9.3** | **6.9** | **2.1** | **1.8** | **1.7** | **2.2** | **1.8** | **2.3** | **2.2** | **0.5** | **2.0** |
|  |  | (1.2–3.0) | (1.3–2.9) | (0.4–3.2) | (3.1–20.3) | (0.8–22.8) | (1.6–2.7) | (1.0–3.2) | (0.9–3.1) | (0.6–5.6) | (1.2–2.7) | (1.1–4.3) | (1.2–3.7) | (0.01–3.0) | (1.3–3.0) |
| III | TCY ^f^ | **3.6** | **4.7** | **2.2** | **50.0** | **13.8** | **4.9** | **3.7** | **2.5** | **3.9** | **3.2** | **3.6** | **6.9** | **0.5** | **4.8** |
|  |  | (2.6–5.0) | (3.6–6.0) | (1.0–4.3) | (36.1–63.9) | (3.9–31.7) | (4.2–5.8) | (2.4–5.4) | (1.4–4.0) | (1.6–7.9) | (2.4–2.2) | (2.0–6.0) | (5.0–9.2) | (0.01–3.0) | (3.7–6.2) |

^a^  Results are presented at the sample level.

^b^ CI = confidence interval.

^c^ Antimicrobial drugs are listed according to the drug’s importance to human medicine (Category IIII).

Category I—antimicrobials of very high importance in human medicine, essential to the treatment of serious bacterial infections, no alternatives for resistant infections; Category II— antimicrobials of high importance in human medicine, used to treat a variety of infections, alternatives for resistance to category III antimicrobials; Category III— antimicrobials of medium importance in human medicine, used as first-line drugs, alternatives for resistance are generally available (Veterinary Drugs Directorate 2009).

^d^ AMC, amoxicillin-clavulanic acid; AMP, ampicillin; CHL, chloramphenicol ; CIP, ciprofloxacin; CRO, ceftriaxone; FOX, cefoxitin; GEN, gentamicin; KAN, kanamycin; NAL, nalidixic acid; SOX, sulfisoxazole; STR, streptomycin; SXT, trimethoprim-sulfamethoxazole; TCY, tetracycline; and TIO, ceftiofur. Reduced susceptibility for azithromycin was not detected in any sample types.

^e^ *n* = number *E. coli* positive samples with reduced susceptibility.

^f^ Intermediate resistance detected: AMC, 5 fecal, 1 paw, 10 soil, and 1 lagoon sample; AMP, 1 fecal, 1 paw; and 10 soil samples; CHL, 5 fecal, 4 paw; 9 soil, and 1 dumpster sample; CIP, 2 fecal and 5 soil samples; FOX, 18 fecal, 5 paw, and 19 soil samples; KAN, 2 soil samples; TCY, 1 fecal, 4 soil, and 1 dumpster sample

^g^ Dash indicates resistance was not detected.
