# Supplementary material for: Epidemiology of Antimicrobial Resistance in Escherichia coli Isolates from Raccoons (Procyon lotor) and the Environment on Swine Farms and Conservation Areas in Southern Ontario
Source: PLoS One. 2016 Nov 9;11(11):e0165303. doi: 10.1371/journal.pone.0165303 (PMC5102455; doi:10.1371/journal.pone.0165303)
Supplement: S4 Table — (DOCX) [file pone.0165303.s004.docx]

**TABLE S4. Multi-level univariable models for the most common antimicrobial drugs resistance was detected in *E. coli***

**isolates from raccoon fecal and soil samples.**

|  |  | **Raccoon Fecal Samples ^a^**  (*n* = 1044) ^b^ | | |  | | |  | **Soil Samples ^a^**  (*n* = 2000) ^b^ | | |  | | |
| --- | --- | --- | --- | --- | --- | --- | --- | --- | --- | --- | --- | --- | --- | --- |
| **Drug** | **Category** |  |  |  | **Variance [VPC] ^c^** | | |  |  |  |  | **Variance [VPC]** | | |
|  |  |  |  |  | **(95% CI) ^d^** | | |  |  |  |  | **(95% CI)** | | |
|  |  | **OR ^e^** | **95% CI** | ***P*** | **Site-level** | **Animal-level** | **Sample-level** |  | **OR** | **95% CI** | ***P*** | **Site-level** | **Sample-level** | **Isolate-level** |
| AMP | Juvenile | 0.78 | 0.34–1.82 | 0.571 | **—** ^f^ | 0.64 [16.4] | [83.6] |  | **—** ^g^ | **—** | **—** | **—** | **—** | **—** |
|  | (Adult REF) |  |  |  |  | (0.01–34.03) |  |  |  |  |  |  |  |  |
|  | Male | 1.22 | 0.58–2.60 | 0.596 | **—** ^f^ | 0.72 [18.0] | [82.0] |  | **—** | **—** | **—** | **—** | **—** | **—** |
|  | (Female REF) |  |  |  | **—** ^f^ | (0.02–26.33) |  |  |  |  |  |  |  |  |
|  | August to November | 0.53 | 0.24–1.18 | 0.122 | **—** ^f^ | 0.82 [20.0] | [80.0] |  | 1.02 | 0.46–2.24 | 0.960 | **—** ^f^ | 3.46 [51.2] | [48.8] |
|  | (May to July REF) |  |  |  | **—** ^f^ | (0.03–25.03) |  |  |  |  |  |  | (1.12–10.66) |  |
|  | Swine Farm | 0.67 | 0.30–1.52 | 0.336 | **—** ^f^ | 0.70 [17.6] | [82.4] |  | 1.46 | 0.67–3.16 | 0.336 | **—** ^f^ | 3.34 [50.4] | [49.6] |
|  | (Conservation REF) |  |  |  | **—** ^f^ | (0.17–28.51) |  |  |  |  |  | **—** ^f^ | (1.04–10.78] |  |
|  | Year 2012 ^f^ | 0.51 | 0.21–1.27 | 0.149 | **—** ^f^ | 0.78 [19.2] | [80.8] |  | 1.33 | 0.50–3.53 | 0.566 | **—** ^f^ | 3.50 [51.6] | [48.4] |
|  | (2011 REF) |  |  |  | **—** ^f^ | (0.02–24.37) |  |  |  |  |  | **—** ^f^ | (1.12–10.88) |  |
|  | Year 2013 | 0.76 | 0.31–1.88 | 0.557 | **—** ^f^ | 0.78 [19.2] | [80.8] |  | 0.72 | 0.26–1.96 | 0.521 | **—** ^f^ | 3.50 [51.6] | [48.4] |
|  | (2011 REF) |  |  |  |  | (0.02–24.37) |  |  |  |  |  |  | (1.12–10.88) |  |
|  |  |  |  |  |  |  |  |  |  |  |  |  |  |  |
| FOX | Juvenile | 0.67 | 0.26–1.72 | 0.403 | 0.67 [8.5] | 0.04 [1.0] | [90.5] |  | **—** | **—** | **—** | **—** | **—** | **—** |
|  | (Adult REF) |  |  |  | (0.02–3.86) | (6.13 x 10^-31^– 2.1 x 10^27^ |  |  |  |  |  |  |  |  |
|  | Male | 0.87 | 0.39–1.96 | 0.743 | 0.29 [8.0] | 0.06 [1.6] | [90.4] |  | **—** | **—** | **—** | **—** | **—** | **—** |
|  | (Female REF) |  |  |  | (0.02–4.13) | (1.1 x 10^-20^– 2.8 x 10^17^ |  |  |  |  |  |  |  |  |
|  | August to November | 1.74 | 0.77–3.96 | 0.185 | 0.26 [7.2] | 0.07 [2.0] | [90.8] |  | 1.75 | 0.87–3.55 | 0.116 | **—** ^f^ | **—** ^f^ | **—** |
|  | (May to July REF) |  |  |  | (0.02–4.43) | (5.4 x 10^-16^– 1.0 x 10^13^) |  |  |  |  |  |  |  |  |
|  | Swine Farm | 0.42 | 0.14–1.22 | 0.111 | 0.09 [2.7] | **—** ^f^ | [97.3] |  | 1.30 | 0.69–2.47 | 0.416 | **—** ^f^ | **—** ^f^ | **—** |
|  | (Conservation REF) |  |  |  | (3.4 x 10^-4^–24.17) |  |  |  |  |  |  |  |  |  |
|  | **Year 2012 ^f^** | **0.25** | **0.09–0.71** | **0.009** | **0.51[13.5]** | **—** ^f^ | **[86.5]** |  | **0.23** | **0.05–0.96** | **0.044** | **—** ^f^ | **—** ^f^ | **—** |
|  | **(2011 REF)** |  |  |  | **(0.06–4.21)** |  |  |  |  |  |  |  |  |  |
|  | **Year 2013** | **0.24** | **0.07–0.80** | **0.021** | **0.51[13.5]** | **—** ^f^ | **[86.5]** |  | **0.23** | **0.71–0.76** | **0.016** | **—** ^f^ | **—** ^f^ | **—** |
|  | **(2011 REF)** |  |  |  | **(0.06–4.21)** |  |  |  |  |  |  |  |  |  |
|  | Year 2013 | 0.96 | 0.26–3.47 | 0.946 | 0.51[13.5] | **—** ^f^ | [86.5] |  | 1.00 | 0.17**–**6.14 | 0.983 | **—** ^f^ | **—** ^f^ | **—** |
|  | (2012 REF) |  |  |  | (0.06–4.21) |  |  |  |  |  |  |  |  |  |
| SOX | Juvenile | 0.74 | 0.26–2.09 | 0.570 | 0.44 [11.6] | **—** ^f^ | [88.4] |  | **—** | **—** | **—** | **—** | **—** ^f^ | **—** |
|  | (Adult REF) |  |  |  | (0.05–4.13) |  |  |  |  |  |  |  |  |  |
|  | Male | 1.30 | 0.54–3.12 | 0.556 | 0.47 [12.6] | **—** ^f^ | [87.4] |  | **—** | **—** | **—** | **—** | **—** | **—** |
|  | (Female REF) |  |  |  | (0.05–4.11) |  |  |  |  |  |  |  |  |  |
|  | August to November | 0.98 | 0.39–2.42 | 0.960 | 0.45 [12.0] | **—** ^f^ | [88.0] |  | 0.58 | 0.23–1.48 | 0.256 | **—** ^f^ | 3.45 [51.2] | [48.8] |
|  | (May to July REF) |  |  |  | (0.05–4.16) |  |  |  |  |  |  |  | (0.85–14.02) |  |
|  | Swine Farm | 1.16 | 0.32–4.10 | 0.824 | 0.43[11.5] | **—** ^f^ | [88.5] |  | 1.40 | 0.57–3.40 | 0.464 | **—** ^f^ | 3.08 [48.4] | [51.6] |
|  | (Conservation REF) |  |  |  | (0.04–4.28) |  |  |  |  |  |  |  | (0.65**–**14.60) |  |
|  | Year 2012 ^f^ | 1.37 | 0.43–4.38 | 0.594 | 0.40[10.8] | **—** ^f^ | [89.2] |  | 1.35 | 0.43–4.26 | 0.608 | **—** ^f^ | 3.08 [48.3] | [51.7] |
|  | (2011 REF) |  |  |  | (0.04–4.22) |  |  |  |  |  |  |  | (0.67**–**14.03) |  |
|  | Year 2013 | 1.21 | 0.35–4.16 | 0.760 | 0.40[10.8] | **—** ^f^ | [89.2] |  | 1.07 | 0.37–3.12 | 0.895 | **—** ^f^ | 3.08 [48.3] | [51.7] |
|  | (2011 REF) |  |  |  | (0.04–4.22) |  |  |  |  |  |  |  | (0.67**–**14.03) |  |
|  |  |  |  |  |  |  |  |  |  |  |  |  |  |  |
| STR | Juvenile | 0.60 | 0.20–1.82 | 0.366 | **—** ^f^ | **—** ^f^ | **—** |  | **—** | **—** | **—** | **—** | **—** | **—** |
|  | (Adult REF) |  |  |  |  |  |  |  |  |  |  |  |  |  |
|  | Male | 1.25 | 0.50–3.10 | 0.631 | **—** ^f^ | **—** ^f^ | **—** |  | **—** | **—** | **—** | **—** | **—** | **—** |
|  | (Female REF) |  |  |  |  |  |  |  |  |  |  |  |  |  |
|  | August to November | 0.41 | 0.15–1.16 | 0.092 | **—** ^f^ | **—** ^f^ | **—** |  | 0.95 | 0.36–2.49 | 0.923 | 0.15 [1.6] | 5.80 [62.8] | [35.6] |
|  | (May to July REF) |  |  |  |  |  |  |  |  |  |  | 7.0 x 10^-4^**–**28.05 | (2.42–13.90) |  |
|  | **Swine Farm** | 1.89 | 0.76–4.70 | 0.169 | **—** ^f^ | **—** ^f^ | **—** |  | **2.95** | **1.02–8.50** | **0.045** | **—** ^f^ | **5.94 [64.3]** | **[35.7]** |
|  | **Conservation REF)** |  |  |  |  |  |  |  |  |  |  |  | **(2.47–14.28)** |  |
|  | Year 2012 ^f^ | 1.18 | 0.41–3.34 | 0.761 | **—** ^f^ | **—** ^f^ | **—** |  | 2.31 | 0.72–7.44 | 0.159 | 0.06 [0.7] | 5.36 [61.6] | [37.8] |
|  | (2011 REF) |  |  |  |  |  |  |  |  |  |  | (2.1 x 10^-6^**–**1586.64) | (2.27–12.70) |  |
|  | Year 2013 | 0.74 | 0.21–2.66 | 0.647 | **—** ^f^ | **—** ^f^ | **—** |  | 1.84 | 0.63–5.38 | 0.264 | 0.06 [0.7] | 5.36 [61.6] | [37.8] |
|  | (2011 REF) |  |  |  |  |  |  |  |  |  |  | (2.1 x 10^-6^**–**1586.64) | (2.27–12.70) |  |
|  |  |  |  |  |  |  |  |  |  |  |  |  |  |  |
| TCY | Juvenile | 0.48 | 0.19–1.17 | 0.106 | 0.10 [2.1] | 1.22 [26.4] | [71.5] |  | **—** | **—** | **—** | **—** | **—** | **—** |
|  | (Adult REF) |  |  |  | (0.01–6.31) | (0.19–8.01) |  |  |  |  |  |  |  |  |
|  | Male | 1.25 | 0.59 –2.63 | 0.564 | 0.07 [1.5] | 1.46 [30.3] | [68.2] |  | **—** | **—** | **—** | **—** | **—** | **—** |
|  | (Female REF) |  |  |  | (3.5 x 10^-4^–15.82) | (0.28–7.55) |  |  |  |  |  |  |  |  |
|  | August to November | 0.50 | 0.24–1.07 | 0.075 | 0.06 [1.2] | 1.42 [29.9] | [68.9] |  | 1.01 | 0.52–1.96 | 0.975 | 0.63 [8.7] | 3.39 [46.3] | [45.0] |
|  | (May to July REF) |  |  |  | (1.0 x 10^-4^–1.43) | (0.25–8.03) |  |  |  |  |  | (0.26–2.56) | (1.17–9.81) |  |
|  | **Swine Farm** | 1.04 | 0.46–2.37 | 0.918 | 0.06[1.2] | 1.40 [29.5] | [69.2] |  | **3.84** | **1.51–9.80** | **0.005** | **0.17 [2.5]** | **3.45 [49.9]** | **[47.6]** |
|  | **(Conservation REF)** |  |  |  | (1.0 x 10^-5^–7.49) | (0.26–7.49) |  |  |  |  |  | **(0.15–2.03)** | **(1.18–10.09)** |  |
|  | Year 2012 ^f^ | 0.68 | 0.29–1.59 | 0.373 | 0.07 [1.4] | 1.43 [29.9] | [68.7] |  | 1.01 | 0.40–2.58 | 0.982 | 0.54 [7.7] | 3.18 [45.4] | [47.0] |
|  | (2011 REF) |  |  |  | (1.8 x 10^-4^–26.19) | (0.27–7.65) |  |  |  |  |  | (0.12–2.34) | (1.10–9.20) |  |
|  | Year 2013 | 0.86 | 0.35–2.11 | 0.748 | 0.07 [1.4] | 1.43 [29.9] | [68.1] |  | 1.62 | 0.75–3.48 | 0.218 | 0.54 [7.7] | 3.18 [45.4] | [47.0] |
|  | (2011 REF) |  |  |  | (1.8 x 10^-4^–26.19) | (0.27–7.65) |  |  |  |  |  | (0.12–2.34) | (1.10–9.20) |  |

^a^ Significant differences are highlighted in bold.

^b^ n = total number of observations in the model

^c^ VPC = variance partition coefficient

^d^ CI = confidence interval

^e^ OR = odds ratio

^f^ For raccoon fecal samples, Wald’s χ^2^ test for year was 0.352 for AMP, 0.012 for FOX, 0.867 for SOX, 0.749 for STR, and 0.667 for TCY. For soil samples, Wald’s χ^2^ test for year was 0.601 for AMP, 0.0099 for FOX, 0.877 for SOX, 0.305 for STR, and 0.421 for TCY.

^g^ Random effect was removed from the analysis because it explained 9.0 x 10^-53^ to 2.0 x 10^-17^ of the variance and its removal had little to no effect on the coefficients and slightly improved or did not change model fit based on AIC and BIC.

^h^ Dashes indicate not applicable.
