# Supplementary material for: Epidemiology of Antimicrobial Resistance in Escherichia coli Isolates from Raccoons (Procyon lotor) and the Environment on Swine Farms and Conservation Areas in Southern Ontario
Source: PLoS One. 2016 Nov 9;11(11):e0165303. doi: 10.1371/journal.pone.0165303 (PMC5102455; doi:10.1371/journal.pone.0165303)
Supplement: S5 Table — (DOCX) [file pone.0165303.s005.docx]

**TABLE S5. Percentage (95% CI) of antimicrobial resistance genes in resistant *E. coli* isolates for all sample types overall and**

**on conservation and swine farms in southern Ontario.**

|  | **% (95% CI) Overall ^a, b, c^** | | | | |  | **% (95% CI) Conservation Area ^a, b, c^** | | |  | **% (95% CI) Swine Farm ^a, b, c^** | | |  |
| --- | --- | --- | --- | --- | --- | --- | --- | --- | --- | --- | --- | --- | --- | --- |
| **Genes ^d^** | **Feces** | **Soil** | **Paws** | **Manure pit** | **Dumpster** | **Total** | **Feces** | **Soil** | **Paws** | **Total** | **Feces** | **Soil** | **Paws** | **Total** |
|  | [*n*=77] ^e^ | [*n*=121] | [*n*=23] | [*n*=31] | [*n*=4] | [*n*=256] | [*n*=51] | [*n*=48] | [*n*=17] | [n=116] | [*n*=26] | [*n*=73] | [n=6] | [*n*=105] |
| **Sulfonamides** |  |  |  |  |  |  |  |  |  |  |  |  |  |  |
| *sul1* | **13.0** | **6.6** | **8.7** | **9.7** | **— ^f^** | **9.0** | **9.8** | **8.3** | **5.9** | **7.8** | **19.2** | **5.5** | **16.7** | **8.6** |
|  | (6.4–22.6) | (2.9–12.6) | (1.1–28.0) | (2.0–25.8) |  | (5.8–13.2) | (3.3–21.4) | (2.3–20.0) | (0.1–28.7) | (3.6–14.2) | (6.6–39.4) | (1.5–13.4) | (0.4–64.1) | (4.0–15.6) |
| *sul2* | **16.9** | **14.0** | **8.7** | **3.2** | **25.0** | **13.3** | **13.7** | **14.6** | **11.8** | **13.8** | **23.1** | **13.7** | **—** | **15.2** |
|  | (9.3–27.1) | (8.4–21.5) | (1.1–28.0) | (0.1–16.7) | (0.6–80.1) | (9.4–18.1) | (5.7–26.3) | (6.1–27.8) | (1.4–36.4) | (8.1–21.4) | (9.0–43.6) | (6.8–23.8) |  | (9.0–23.6) |
| *sul3* | **1.3** | **1.7** | **8.7** | **3.2** | **25.0** | **2.0** | **—** | **—** | **11.8** | **1.7** | **3.8** | **2.7** | **—** | **2.9** |
|  | (0.03–7.0) | (0.2–5.8) | (1.1–28.0) | (0.1–16.7) | (0.6–80.1) | (0.6–4.5) |  |  | (1.4–36.4 | (0.2–6.1) | (0.1–19.6) | (0.3–9.5) |  | (0.6–8.1) |
| **Tetracyclines** |  |  |  |  |  |  |  |  |  |  |  |  |  |  |
| *tet* (A) | **28.6** | **28.1** | **17.4** | **38.7** | **75.0** | **29.3** | **25.5** | **18.8** | **17.6** | **21.6** | **34.6** | **34.2** | **16.7** | **33.3** |
|  | (18.8–40.0) | (20.3–40.0) | (5.0–38.8) | (21.8–57.8) | (19.4–99.4) | (23.8–35.3) | (14.3–39.6) | (8.9–32.6) | (3.8–43.4) | (14.4–30.1) | (17.2–55.7) | (23.5–46.3) | (0.4–64.1) | (24.4–43.2) |
| *tet* (B) | **15.6** | **17.4** | **13.0** | **48.4** | **—** | **20.3** | **15.7** | **12.5** | **17.6** | **14.6** | **19.2** | **20.5** | **—** | **19.0** |
|  | (8.3–25.6) | (11.1–25.3) | (2.8–33.6) | (30.2–66.9) |  | (15.6–25.8) | (7.0–28.6) | (4.7–25.2) | (3.8–43.4) | (8.9–22.4) | (6.6–39.4) | (12.0–31.6) |  | (12.0–27.9) |
| *tet* (C) | **1.3** | **5.8** | **8.7** | **—** | **—** | **3.9** | **1.8** | **4.2** | **11.8** | **4.3** | **—** | **6.8** | **—** | **4.8** |
|  | (0.03–7.0) | (2.4–11.6) | (1.1–28.0) |  |  | (1.9–7.1) | (0.05–10.4) | (0.5–14.2) | (1.4–36.4) | (1.4–9.8) |  | (2.3–15.3) |  | (1.6–10.8) |
| **Betalactams** |  |  |  |  |  |  |  |  |  |  |  |  |  |  |
| *bla*_CMY-2_ | **7.8** | **1.7** | **8.7** | **—** | **—** | **3.9** | **9.8** | **4.2** | **11.8** | **7.8** | **3.8** | **—** | **—** | **1.0** |
|  | (2.9–16.2) | (0.2–5.8) | (1.1–28.0) |  |  | (1.9–7.1) | (3.2–21.4) | (0.5–14.2) | (1.4–36.4) | (3.6–14.2) | (0.1–19.6) |  |  | (0.02–5.2) |
| *bla*_TEM_ | **27.3** | **13.2** | **13.0** | **29.0** | **—** | **19.1** | **25.4** | **10.4** | **17.6** | **18.1** | **30.8** | **15. 1** | **—** | **18.1** |
|  | (17.7–38.6) | (7.8–20.6) | (2.8–33.6) | (14.2–48.0) |  | (14.5–24.5) | (14.3–39.6) | (3.5–22.7) | (3.8–43.4) | (11.6–26.3) | (14.3–51.8) | (7.8–25.4) |  | (11.3–26.8) |
| *bla*_CTX-M_ | **1.3** | **—** | **—** | **—** | **—** | **0.4** | **1.8** | **—** | **—** | **0.9** | **—** | **—** | **—** | **—** |
|  | (0.03–7.0) |  |  |  |  | (0.01–2.2) | (0.05–10.4) |  |  | (0.02–4.7) |  |  |  |  |
| **Aminoglycosides** |  |  |  |  |  |  |  |  |  |  |  |  |  |  |
| *aphA1* | **3.9** | **5.0** | **4.3** | **—** | **—** | **3.9** | **3.7** | **8.3** | **5.9** | **6.0** | **3.8** | **2.7** | **—** | **2.9** |
|  | (0.8–11.0) | (1.8–10.5) | (0.1–21.9) |  |  | (1.9–7.1) | (0.5–13.5) | (2.3–20.0) | (0.1–28.7) | (2.5–12.0) | (0.1–19.6) | (0.3–9.5) |  | (0.6–8.1) |
| *aphA2* | — | — | — | **3.2** | — | **0.4** | — | — | — | **0.9** | — | — | — | **—** |
|  |  |  |  | (0.1–16.7) |  | (0.01–2.2) |  |  |  | (0.02–4.7) |  |  |  |  |
| *strAB* | **20.8** | **24.8** | **8.7** | **41.9** | **25.0** | **24.2** | **15.7** | **16.7** | **5.9** | **14.7** | **30.8** | **30.1** | **16.7** | **29.5** |
|  | (12.4–31.5) | (17.4–33.5**)** | (1.1–28.0) | (24.5–60.9) | (0.6–80.1) | (19.1–29.9) | (7.0–28.6) | (7.5–30.2) | (0.1–28.7) | (8.9–22.4) | (14.3–51.8) | (20.0–42.0) | (0.4–64.1) | (21.0–39.2) |
| *aadA* | **13.0** | **13.2** | **13.0** | **22.6** | **25.0** | **14.4** | **7.8** | **14.6** | **11.8** | **11.2** | **23.1** | **12.3** | **16.7** | **15.2** |
|  | (6.4–22.6) | (7.8–20.6) | (2.8–33.6) | (9.6–41.1) | (0.6–80.1) | (10.4–19.4) | (2.2–18.9) | (6.1–27.8) | (1.4–36.4) | (6.1–18.4) | (9.0–43.6) | (5.8–22.1) | (0.4–64.1 | (9.0–23.6) |
| **Phenicols** |  |  |  |  |  |  |  |  |  |  |  |  |  |  |
| *catA1* | **2.6** | **—** | **—** | **—** | **25.0** | **1.2** | **3.9** | **—** | **—** | **1.7** | **—** | **—** | **—** | **—** |
|  | (0.3–9.1) |  |  |  | (0.6–80.1) | (0.2–3.4) | (0.5–13.5) |  |  | (0.2–6.1) |  |  |  |  |
| *cmlA* | **1.3** | **0.8** | **8.7** | **3.2** | **25.0** | **2.3** | **—** | **—** | **11.8** | **1.7** | **3.8** | **1.4** | **—** | **1.9** |
|  | (0.03–7.0) | (0.02–4.5) | (1.1–28.0) | (0.1–16.7) | (0.6–80.1) | (0.9–5.0) |  |  | (1.4–36.4) | (0.2–6.1) | (0.1–19.6) | (0.03–7.4) |  | (0.2–6.7) |
| *floR* | **6.5** | **2.5** | **—** | **3.2** | **—** | **3.5** | **3.9** | **—** | **—** | **1.7** | **11.5** | **4.1** | **—** | **5.7** |
|  | (2.1–14.5) | (0.5–7.1) |  | (0.1–16.7) |  | (1.6–6.6) | (0.5–13.5) |  |  | (0.2–6.1) | (2.4–30.2) | (0.9–11.5) |  | (2.1–12.0) |
|  |  |  |  |  |  |  |  |  |  |  |  |  |  |  |

^a^ Results are presented at the sample level.

^b^ Isolates classified as having intermediate susceptibility were classified as being resistant.

^c^ CI = confidence interval.

^d^ Resistance genes for *aadB,* *aac(3)IV, bla*_SHV_, and *bla*_OXA1_ were not detected in any sample types.

^e^ *n* = Number *E. coli* positive samples with reduced susceptibility.

^f^ Dash indicates no resistance genes detected.
